# Research and Remedies

**Published:** 1914-02-28

**Authors:** 


### RESEARCH AND REMEDIES.

### The Prediction of Sex.

A good deal has been heard the last year or two, chiefly in America and on the Continent, of endeavours to diagnose pregnancy by making use of blood reactions of the Bordet-Gengou type, upon which the Wassermann test, to mention one out of many, is based. So far it has not been proved that any of these attempts have succeeded, not even the best known, that of Abderhalden, though it is not by any means impossible or improbable that success will come some day. Pankow has created some stir by an ingenious account (in the *Zeitschrift f. Geburtsch. und Gyn.*) of his experiments to determine the sex of the unborn child during the last month of pregnancy. The hypothesis underlying his methods is that the fœtal testes, being glandular organs with an internal secretion as well as an external, may secrete substances which could be demonstrated in the blood of a woman pregnant with a male child; whereas the absence of such substances would indicate a female child. His attempts to distinguish such a hypothetical substance were first made on animals, and were very patiently worked out. He thinks there is reason to believe that his hypothetical substances exist, though he admits at present his inability to detect them with accuracy. The methods used for the determination were of the complement fixation type, and he looks forward to ultimate success.

### The Treatment of Actinomycosis.

In the *Boston Medical and Surgical Journal* Kinnicutt and Mixter report their attempts to produce a vaccine which shall benefit those suffering from actinomycosis. Empirically they grew the fungus on various media so as to obtain a pure culture, and having done this they let the organism grow for a week to ten days. Then they ground up the colonies in a sterile tube, added normal saline, and heated the result at 60° C. for one hour. After this the vaccine was transferred to a bottle containing more (sterile) saline solution and also a quarter per cent. of lysol. Very small doses were used to begin with, and were given in gradually increased quantity every three or four days. Local treatment in the way of drainage of all abscesses was carried on at the same time. Out of four cases in which the jaws and neck were involved, three ended in complete recovery and one in great improvement. Two cases of advanced actinomycosis of the lung showed no improvement. One abdominal case is apparently cured, and another has terminated fatally. The results, as far as they go, are encouraging; and it is to be hoped that others will confirm these workers.

### Pellagra and Sprue.

The discovery of a good many cases of pellagra in British mental hospitals, and the not long previous realisation of its tremendous ravages in the Southern States of the U.S.A., make this disease and its relations exceedingly important to institutional medical officers. Quite lately the idea has been put forward that it is identical with sprue, a tropical and sub-tropical ailment which does not occur, so far as is known, in Britain. The difficulty of this identification consists in the fact that the skin eruptions and the mental disturbances characteristic of pellagra are not seen in sprue; in other respects the symptoms are very similar. It is now being argued that neither skin lesions nor insanity are essential symptoms of pellagra; some patients have the former without the latter, and some the latter without the former. It is, therefore, conceivable that patients may have pellagra and yet display neither of these signs. Stomatitis and other digestive disturbances are an absolutely constant feature of both diseases; and both at the bedside and in the laboratory these changes are practically the same. When the pathology and ætiology of pellagra are more settled than at present, it will be possible for the experts to pronounce on this interesting point about the identity of sprue (psilosis) and pellagra.

